# Renin-Angiotensin System Inhibitors, Type 2 Diabetes and Fibrosis Progression: An Observational Study in Patients with Nonalcoholic Fatty Liver Disease

**DOI:** 10.1371/journal.pone.0163069

**Published:** 2016-09-20

**Authors:** Serena Pelusi, Salvatore Petta, Chiara Rosso, Vittorio Borroni, Anna Ludovica Fracanzani, Paola Dongiovanni, Antonio Craxi, Elisabetta Bugianesi, Silvia Fargion, Luca Valenti

**Affiliations:** 1 Internal Medicine and Metabolic Diseases, Fondazione IRCCS Ca’ Granda Ospedale Maggiore Policlinico, Università degli Studi di Milano, Milano, Italy; 2 Gastroenterology, Università di Palermo, Palermo, Italy; 3 Gastroenterology, Dept. of Medical Sciences, Università di Torino, Torino, Italy; The Chinese University of Hong Kong, HONG KONG

## Abstract

**Background:**

The clinical determinants of fibrosis progression in nonalcoholic fatty liver disease (NAFLD) are still under definition.

**Aim:**

To assess the clinical determinants of fibrosis progression rate (FPR) in NAFLD patients with baseline and follow-up histological evaluation, with a special focus on the impact of pharmacological therapy.

**Methods:**

In an observational cohort of 118 Italian patients from tertiary referral centers, liver histology was evaluated according to Kleiner. Independent predictors of FPR were selected by a stepwise regression approach.

**Results:**

Median follow-up was 36 months (IQR 24–77). Twenty-five patients (18%) showed some amelioration, 63 (53%) had stability, 30 (25%) had progression of fibrosis. Patients with nonalcoholic steatohepatitis (NASH) had similar demographic and anthropometric features, but a higher prevalence of type 2 diabetes (T2D; p = 0.010), and use of renin-angiotensin axis system (RAS) inhibitors (p = 0.005). Fibrosis progression was dependent of the length of follow-up, and was associated with, but did not require, the presence of NASH (p<0.05). Both fibrosis progression and faster FPR were independently associated with higher APRI score at follow-up, absence of treatment with RAS inhibitors, and T2D diagnosis at baseline (p<0.05). There was a significant interaction between use of RAS inhibitors and T2D on FPR (p = 0.002). RAS inhibitors were associated with slower FPR in patients with (p = 0.011), but not in those without (p = NS) T2D.

**Conclusions:**

NASH is not required for fibrosis progression in NAFLD, whereas T2D seems to drive fibrogenesis independently of hepatic inflammation. Use of RAS inhibitors may contrast fibrosis progression especially in high-risk patients affected by T2D.

## Introduction

Nonalcoholic fatty liver disease (NAFLD) is commonly held as the hepatic manifestation of obesity and insulin resistance. Due to the worldwide epidemics of obesity and type 2 diabetes (T2D), NAFLD is projected to become the leading cause of hepatocellular carcinoma and end-stage liver disease within the next ten years[1]. Despite NAFLD affects nearly one third of the population, progressive liver disease remains a relatively rare complication of this condition[1]. Cross-sectional studies have identified severity of overweight, T2D, muscle fitness, dietary factors, lack of use of lipid lowering drugs such as statins, and genetic predisposition as risk factors for advanced disease [2–5]. However, the clinical determinants of progression of fibrosis, the main determinant of liver-related outcomes and overall mortality[6,7], are still under definition. Indeed, data from prospective studies are still very limited[8,9]. Overall evidence suggests that when steatosis is associated with hepatocellular damage and necroinflammation, that is nonalcoholic steatohepatitis (NASH), higher AST/ALT ratio, and in the presence of hyperglycemia, fibrosis progression rate (FPR) is faster[8–10]. Yet, some individuals with simple steatosis have fast-progressing disease, especially when gain weight or develop T2D [9,11]. Furthermore, arterial hypertension has also been associated with faster FPR[12]. This suggests that neuro-hormonal alterations associated with this condition, and in particular activation of the renin-angiotensin system (RAS), directly favors steatosis, inflammation and fibrogenesis via enhanced activation of hepatic stellate cells, whereas RAS inhibits contrast this process[13–20]. In keeping, RAS inhibitors such as ACE-inhibitors or angiotensin receptor blockers have been associated with improvement of liver damage[21], even if evidence is controversial[22]. Furthermore, in cross-sectional studies RAS inhibition protected from severe fibrosis in patients with hypertension and NAFLD[23], and was associated with reduced liver stiffness in patients with chronic kidney disease [24]

Aim of this study was therefore to assess the clinical determinants of FPR in an ethnically homogeneous cohort of Italian patients with histological diagnosis of NAFLD, with a special focus on the impact of pharmacological therapy.

## Methods

### Patients

In the study retrospective data collected from 118 consecutive patients from Italian ancestry with clinical and histological diagnosis of NAFLD were prospectively evaluated. Patients were followed-up at three tertiary referral centers in Italy (Milan, n = 67, 57%, Palermo, n = 32, 27%, and Turin, n = 19, 16%), for whom a baseline and a follow-up liver biopsy and clinical data were available between January 1992 and June 2015.

In all patients other liver diseases were ruled out by standard assessment[2,25], and alcohol intake (evaluated by a questionnaire) had to be lower than 30/20 g/day in males/females, respectively. Patients with decompensated cirrhosis, hepatocellular carcinoma, and current use of steatosis inducing drugs were also excluded.

In all subjects, first biopsy was performed for suspected NASH in the presence of persistently elevated liver enzymes, or a long history of NAFLD associated with severe insulin resistance. Follow-up control biopsy was routinely offered to all compliant patients at five years, or indicated when alterations in the clinical picture or imaging suggested progressive liver disease. We also included patients randomized to iron depletion [26] or vitamin D supplementation (http://www.webaisf.org/studi-e-ricerche/studi-in-corso.aspx) vs. lifestyle changes alone in open label trials, as these treatments were not demonstrated to influence fibrosis progression. Patients randomized to active arms in pharmacological studies, where the investigational product was shown to improve liver histology, or who underwent bariatric surgery procedures between the two biopsies (n = 13) were excluded.

The study was carried out in accordance with the principles of the Helsinki Declaration, and with local and national laws. Approval was obtained from the hospital Internal Review Boards and Ethics Committees of the Fondazione IRCCS Ca’ Granda Ospedale Maggiore Policlinico Milano, Azienda Ospedaliera Universitaria Citta´ della Salute e della Scienza Torino and Azienda Ospedaliera Universitaria Policlinico Palermo and written informed consent was obtained from all patients. Clinical and laboratory assessment is described in details in the Supplementary methods.

### Histological analysis

Slides were coded and read by one expert pathologist at each center, who was unaware of patients’ identity and history. A minimum 15mm-length of the biopsy specimen or the presence of at least 10 complete portal tracts was required[27]. Clinically significant steatosis was defined as steatosis involving ≥5% of hepatocytes[28]. Diagnosis of NASH was based on the presence of steatosis with both lobular necroinflammation and ballooning[28,29]. Disease activity and fibrosis stage were assessed according to the NAFLD activity score (NAS) and staging[28]. We previously observed a good correlation for liver fibrosis assessment among the centers involved in the study[30].

### Statistical analysis

For descriptive statistics, continuous traits were summarized as means±SD. Highly skewed variables, were summarized as medians and interquartile range, and log-transformed before analysis. Categorical variables are shown as percentages. Baseline and follow-up clinical features of patients were compared by chi-square and paired t-test, as required. Independent predictors of fibrosis progression (increase in at least one stage) were determined by logistic regression, considering as independent variables those significant at univariate analysis. FPR was calculated by taking the ratio between the difference of fibrosis stage and the time (months) between the baseline and follow-up biopsy, and it was treated as a continuous variable. A multivariate regression model with a stepwise regression procedure was set to identify the strongest predictors of FPR, among all variables considered in the study (enlisted in supplementary material). A significance level of 0.1 was defined to allow a variable into the model, and a significance level of 0.25 was defined for a variable to stay into the model. A generalized linear model was then fit to examine the independent predictors of FPR, excluding highly correlated variables to avoid collinearity. In this final model, product terms between variables were evaluated to investigate the interaction between risk factors on FPR.

Statistical analyses were carried out with JMP 12.0 (SAS Institute, Cary, NC) and SPSS 21.0 (IBM, Burbank, NJ). A two-sided P value <0.05 was considered statistically significant.

## Results

### Study cohort and clinical evolution at follow-up

The baseline clinical features of patients included in the study are shown in Table 1, left column. They were mostly middle-aged men or post-menopausal women, overweight or obese, with a high prevalence of metabolic alterations defining metabolic syndrome and/or altered liver enzymes (Table 1). Forty-two % had histological NASH.

**Table 1:** Clinical features of 118 Italian patients with NAFLD, who underwent a follow-up liver biopsy.

Clinical features	Baseline	Follow-up	p value
Sex, F	45 (38)	45 (38)	1.00
Age, years	47±12	51±11	<0.001
BMI, Kg/m^2^	30.6±6.6	29.0±7.2	0.006
T2D, yes	29 (25)	32 (27)	0.66
Glucose, mg/dl	98±25	102±25	0.13
Total cholesterol, mg/dl	194±44	189±43	0.066
HDL cholesterol, mg/dl	47±14	49±13	0.054
Triglycerides, mg/dl	111 {78–161}	111 {73–154}	0.078
Arterial hypertension, yes	38 (32)	54 (46)	0.033
ALT, IU/ml	50 {20–83}	40 {21–60}	0.002
AST, IU/ml	33 {23–51}	27 {21–36}	0.077
GGT, IU/ml	45 {28–80}	35 {19–63}	0.45
Ferritin (ng/mL)	234 {88–506}	138 {73–334}	0.012
Platelets (x10^9/L)	224 ± 71	224 ± 67	0.9
NASH, yes	49 (42)	47 (40)	0.79
APRI score	0.7 ± 1.7	0.4 ± 0.3	0.11
FIB4 score	1.3 ± 1.1	1.3 ± 0.9	0.82
NFS	-1.7 ± 1.7	-1.6 ±1.6	0.79
RAS inhibitors, yes	26 (22)	36 (31)	0.14
Beta-blockers, yes	14 (12)	19 (16)	0.35
Calcium-antagonists, yes	12 (10)	11 (9)	0.81
Diuretics, yes	9 (8)	9 (8)	1.00
Metformin, yes	22 (19)	30 (26)	0.21
Statins, yes	16 (14)	28 (24)	0.045
Omega-3, yes	7 (6)	8 (7)	0.79
Vitamin E, yes	4 (3)	6 (5)	0.52
Iron depletion, yes	0	12 (10)	<0.001
Length of follow-up, months	36 {24–77}		-

Data are shown as mean±SD, frequency (%), median {IQR}, as required. BMI: body mass index; T2D: type 2 diabetes; RAS: renin angiotensin system. Less than three patients (per drug class) were on glitazones, fibrates, and GLP-1 agonists/DPP-4 inhibitors.

At baseline liver biopsy, 16% of patients were on statins, 22% on RAS inhibitors (14 on ACE-inhibitors and 12 on angiotensin receptor blockers). Indication for RAS inhibitors was treatment of hypertension and/or micro-albuminuria in T2D in 14, and treatment of hypertension without T2D in 12. RAS inhibitors were not indicated for prevention of liver fibrosis progression in this cohort.

Median follow-up was 36 months (IQR 24–77, range 7–196), for a total of 6,509 months. Clinical features of patients at follow-up are shown in Table 1, right column. Patients lost on average 1.6 Kg/m^2^ of body mass (p = 0.006), which was associated with reduced ALT levels (p = 0.002). The prevalence of arterial hypertension (p = 0.033), but not that of T2D, increased at follow-up.

At the end of the observation there was an increase in the prevalence of treatment with statins (p = 0.045). An additional 10% of patients had started RAS inhibitors (7 ACE-inhibitors and 3 angiotensin receptor blockers; 2 with T2D, and 8 with hypertension without T2D; p = NS). Twelve patients with baseline hyperferritinemia underwent iron depletion by phlebotomy during follow-up (p<0.001).

### Fibrosis evolution during follow-up

The prevalence of histological NASH did not change significantly at follow-up (Table 1; p = NS), although average NAS score decreased (median 4, IQR 2–5 at baseline vs. 3, IQR 2–5 at follow-up; p = 0.026). Non-invasive fibrosis scores did not significantly change at follow-up (p = NS; Table 1).

Clinical features of patients stratified by the presence of baseline NASH are presented in Table 2. Patients with NASH had a higher prevalence of T2D (p = 0.010), use of RAS inhibitors (p = 0.005), and in the subgroup of patients for whom data were available, of prevalence of *PNPLA3* 148M/M risk genotype (p = 0.023).

**Table 2:** Clinical features associated with presence of NASH at baseline evaluation.

Clinical features	NASH (n = 49)	Non-NASH (n = 69)	p value
Sex, F	21 (43)	24 (35)	0.37
Age, years	48±12	47±12	0.49
BMI, Kg/m^2^	30.4±4.1	31.0±8.0	0.41
T2D, yes	18 (37)	11 (16)	0.010
Glucose, mg/dl	108±34	92±13	0.002
Total cholesterol, mg/dl	190±40	197±46	0.33
HDL cholesterol, mg/dl	47±15	48±15	0.67
Triglycerides, mg/dl	135±77	130±72	0.71
Arterial hypertension, yes	20 (41)	18 (26)	0.091
ALT, IU/ml	57 {41–95}	47 {24–77}	0.86
AST, IU/ml	37 {27–62}	31 {21–40}	0.60
GGT, IU/ml	54 {31–93}	42 {26–72}	0.45
Ferritin (ng/mL)	196 {51–408}	292 {75–523}	0.11
Platelets (x10^9/L)	237 ± 74	217 ± 70	0.16
NASH, yes	49 (42)	47 (40)	0.79
APRI score	0.5 ± 0.3	0.8 ± 2.2	0.37
FIB4 score	1.3 ± 0.8	1.3 ± 1.3	0.82
NFS	-1.7 ± 1.5	-1.7 ±1.5	0.73
RAS inhibitors, yes	17 (35)	9 (13)	0.005
Beta-blockers, yes	7 (14)	7 (10)	0.49
Calcium-antagonists, yes	8 (16)	4 (6)	0.062
Diuretics, yes	4 (8)	5 (7)	0.85
Metformin, yes	16 (33)	6 (9)	0.001
Statins, yes	8 (16)	8 (12)	0.59
Omega-3, yes	4 (8)	3 (4)	0.39
Vitamin E, yes	4 (3)	6 (5)	0.52
Iron depletion, yes	3 (6)	9 (13)	0.22
*PNPLA3* 148 M/M	12/27 (44)	13/62 (21)	0.023
FPR, stage/month	-0.01 ± 0.05	+0.004 ± 0.03	0.080
Follow-up, months	30 {7–144}	38 {9–196}	0.010

Data are shown as mean±SD, frequency (%), median {IQR}, as required. BMI: body mass index; T2D: type 2 diabetes; RAS: renin angiotensin system; FPR: fibrosis progression rate.

Evolution of liver fibrosis according to baseline stage is presented in Table 3, upper panel. Of 118 patients, 25 (18%) showed some amelioration, 63 (53%) had stability, 30 (25%) had progression of fibrosis. Of note, 5 patients had progression to cirrhosis. Mean FPR was -0.002±0.040. Evolution of fibrosis in patients stratified according to NASH at baseline is presented in Table 3, middle and bottom panels, respectively. Fibrosis stage was more severe in patients with NASH at baseline (p<0.001). FPR was non-significantly lower in patients with than in those without baseline NASH (-0.011±0.054 vs. +0.004±0.033; p = 0.056), and it was not associated with presence of necroinflammation without NASH (p>0.5). No single baseline histological feature of liver damage was able to predict FPR (S1 Table).

**Table 3:** Evolution of liver fibrosis by baseline disease stage in 118 Italian patients with NAFLD.

	Overall						
	Follow-up						Total =
Baseline		Stage 0	Stage 1	Stage 2	Stage 3	Stage 4	
	Stage 0	21 (18)	8 (7)	4 (3)	3 (2)	0	36 (31)
	Stage 1	6 (5)	21 (18)	6 (5)	2 (2)	0	35 (30)
	Stage 2	1 (1)	6 (5)	9 (8)	2 (2)	2 (2)	20 (17)
	Stage 3	1 (1)	5 (4)	3 (2)	5 (4)	3 (2)	17 (14)
	Stage 4	0	1 (1)	2 (2)	0	7 (6)	10 (8)
Total =		29 (25)	41 (35)	24 (20)	12 (10)	12 (10)	118
	NASH						
	Follow-up						Total =
Baseline		Stage 0	Stage 1	Stage 2	Stage 3	Stage 4	
	Stage 0	1 (2)	3 (6)	2 (4)	1 (2)	0	7 (14)
	Stage 1	1 (2)	6 (12)	3 (6)	1 (2)	0	11 (22)
	Stage 2	1 (2)	3 (6)	5 (10)	2 (2)	1 (2)	12 (25)
	Stage 3	0	4 (8)	2 (4)	4 (8)	2 (4)	12 (25)
	Stage 4	0	1 (2)	2 (4)	0	4 (8)	7 (14)
Total =		3 (6)	17 (35)	14 (29)	8 (16)	7 (14)	49
	Non-NASH						
	Follow-up						Total =
Baseline		Stage 0	Stage 1	Stage 2	Stage 3	Stage 4	
	Stage 0	20 (18)	5 (7)	2 (3)	2 (2)	0	29 (42)
	Stage 1	5 (5)	15 (18)	3 (5)	1 (2)	0	24 (35)
	Stage 2	1 (1)	3 (5)	4 (8)	0	1 (2)	8 (12)
	Stage 3	1 (1)	1 (4)	1 (2)	1 (4)	1 (2)	5 (7)
	Stage 4	0	0	0	0	3 (6)	3 (4)
Total =		26 (38)	24 (35)	10 (14)	4 (6)	5 (7)	69

### Clinical predictors of fibrosis progression

Baseline and follow-up clinical features of patients without baseline cirrhosis (n = 10) stratified by progression status are presented in Table 4. Progression of fibrosis was associated with length of follow-up (p = 0.027), lower HDL, absence of use of RAS inhibitors at baseline, AST and ALT levels at baseline and follow-up, APRI score and NASH at follow-up (p<0.05). After correction for duration of observation (Table 4), progression was associated with NASH at baseline and follow-up, T2D at baseline and metformin use (likely a proxy of more severe T2D) at follow-up, and AST and APRI score at follow-up (p<0.05). There was a non-significant trend for a protective effect of use of RAS inhibitors. Among patients without NASH at baseline (n = 69), 6 of 15 (43%) progressors developed NASH at follow-up, vs. 8/46 (15%) of non-progressors (p = 0.044).

**Table 4:** Clinical features associated with fibrosis progression at baseline and follow-up evaluation in 108 patients with NAFLD without F4 fibrosis at baseline.

	Non-progressors (n = 78)	Progressors (n = 30)	p value	p value*
Follow-up, months	36 {24–72}	60 {30–120}	0.027	1.00
BASELINE				
Sex, F	27 (35)	11 (37)	0.83	0.28
Age, years	47±11	45±13	0.33	0.87
BMI, Kg/m^2^	30.7±8.0	29.9±8.4	0.55	0.75
T2D, yes	15 (19)	8 (27)	0.43	0.034
Glucose, mg/dl	98±27	98±21	0.98	0.15
Total cholesterol, mg/dl	200±47	188±33	0.16	0.12
HDL cholesterol, mg/dl	49±14	43±13	0.026	0.14
Triglycerides, mg/dl	129±70	137±86	0.75	0.88
Arterial hypertension, yes	25 (32)	6 (20)	0.24	0.40
ALT, IU/ml	47 {26–72}	72 {39–116}	0.024	0.17
AST, IU/ml	30 {23–39}	40 {26–55}	0.037	0.35
GGT, IU/ml	44 {25–80}	45 {32–65}	0.55	0.46
Ferritin (ng/mL)	161 {72–504}	335 {191–543}	0.12	0.35
Platelets (x10^9/L)	229 ± 51	215 ± 75	0.38	0.51
NASH, yes	27 (35)	15 (50)	0.18	0.037
APRI score	0.4 ± 0.3	1.2 ± 3.3	0.072	0.079
FIB4 score	1.1 ± 0.7	1.3 ± 1.3	0.18	0.060
NFS	-1.8 ± 1.5	-1.9 ±1.8	0.92	0.53
RAS inhibitors, yes	20 (26)	2 (7)	0.028	0.059
Beta-blockers, yes	8 (10)	3 (10)	1.00	0.74
Calcium-antagonists, yes	7 (9)	0	0.19	0.99
Diuretics, yes	6 (8)	1 (3)	0.67	0.69
Metformin, yes	11 (14)	6 (20)	0.55	0.14
Statins, yes	10 (13)	3 (10)	1.00	0.98
Omega-3, yes	5 (6)	1 (3)	1.00	0.64
Vitamin E, yes	2 (3)	1 (3)	1.00	0.92
FOLLOW-UP				
Age, years	52±11	51±11	0.81	0.67
BMI, Kg/m^2^	29.0±6.6	27.8±9.0	0.48	0.42
T2D, yes	17 (22)	9 (30)	0.45	0.094
Glucose, mg/dl	98±22	102±24	0.35	0.10
Total cholesterol, mg/dl	191±41	186±48	0.64	0.66
HDL cholesterol, mg/dl	49±12	46±13	0.26	0.37
Triglycerides, mg/dl	111±54	131±78	0.20	0.27
Arterial hypertension, yes	32 (41)	14 (47)	0.66	0.64
ALT, IU/ml	40 {20–52}	53 {29–82}	0.022	0.13
AST, IU/ml	27 {19–33}	35 {24–46}	0.006	0.014
GGT, IU/ml	28 {15–58}	42 {26–62}	0.36	0.29
Ferritin (ng/mL)	119 {70–296}	237 {90–427}	0.077	0.19
Platelets (x10^9/L)	220 ± 74	231 ± 67	0.49	0.40
NASH, yes	22 (28)	17 (57)	0.008	0.012
APRI score	0.3 ± 0.2	0.5 ± 0.4	0.018	0.008
FIB4 score	1.1 ± 0.7	1.5 ± 1.2	0.16	0.053
NFS	-1.9 ± 1.4	-1.8 ±1.7	0.82	0.36
RAS inhibitors, yes	24 (31)	6 (20)	0.34	0.25
Beta-blockers, yes	24 (14)	5 (17)	0.74	0.50
Calcium-antagonists, yes	6 (8)	0	0.18	0.99
Diuretics, yes	6 (8)	1 (3)	0.67	0.69
Metformin, yes	15 (19)	10 (33)	0.14	0.043
Statins, yes	10 (13)	3 (10)	0.60	0.24
Omega-3, yes	2 (3)	3 (10)	0.13	0.54
Vitamin E, yes	2 (3)	1 (3)	1.00	0.37
Iron depletion, yes	11 (14)	1 (3)	0.17	0.14

Data are shown as mean±SD, frequency (%), median {IQR}, as required.

* p value adjusted for duration of observation at logistic regression analysis.

BMI: body mass index; T2D: type 2 diabetes; RAS: renin angiotensin system.

The association of the changes of clinical variables during follow-up with fibrosis progression is shown in S2 Table. None was associated with progression, even if at unadjusted analysis development of new hypertension was associated with progression (8/30, 27% vs. 7/78, 9%; p = 0.028). The noninvasive independent predictors of progression at multivariate logistic regression analysis are shown in S3 Table. Duration of observation, lack of use of RAS inhibitors, baseline NASH, T2D, and follow-up APRI score were associated with the likelihood of progression (p<0.05).

### Independent predictors of FPR

The independent predictors of FPR are shown in Table 5. Among variables selected by stepwise regression, faster FPR was associated with higher APRI score at follow-up (p = 0.005), absence of treatment with RAS inhibitors (p = 0.009), T2D diagnosis at baseline (p = 0.025). There was a significant interaction between use of RAS inhibitors and T2D (p = 0.002).

**Table 5:** Independent predictors of FPR in 118 Italian patients with NAFLD (including 10 with F4 fibrosis at baseline).

FPR predictor	Estimate±SE	p value
APRI at f-up, per unit	+0.04±0.01	0.005
RAS inhibitors at baseline or f-up	-0.012±0.004	0.009
T2D at baseline, yes	+0.010±0.004	0.025
Beta-blockers at baseline or f-up	-0.008±0.005	0.14
BMI variation, Kg/m^2^	+0.001±0.001	0.14
Hypertension at baseline or f-up	+0.006±0.005	0.24

SE: standard error; F-up: follow-up.

**Fig 1:**
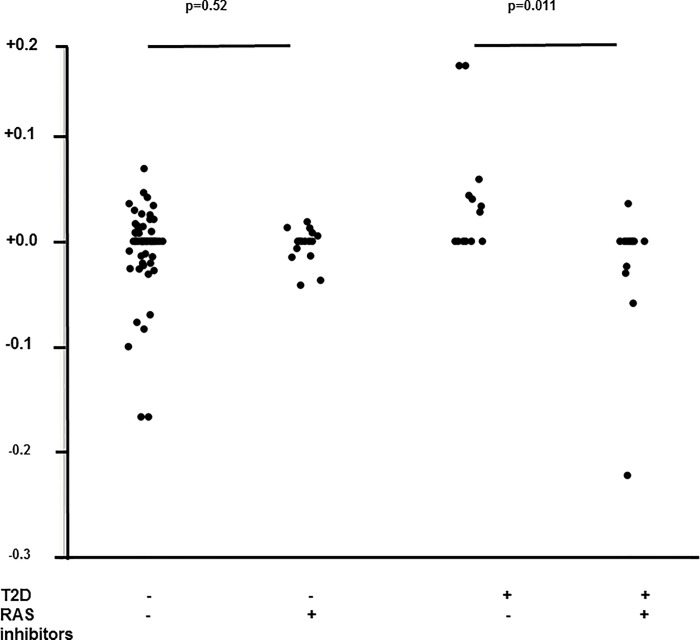
Impact of RAS inhibitors on FPR in patients stratified by the presence of T2D at baseline.

The impact of RAS inhibitors use on FPR in patients stratified by the presence of T2D at diagnosis is shown in Fig 1. RAS inhibitors were associated with slower FPR in patients with (p = 0.011), but not in those without (p = 0.52) T2D.

## Discussion

In this study, we evaluated the clinical determinants of FPR in a relatively large prospective cohort of 118 Italian patients with histological NAFLD, with a special focus on the impact of pharmacological therapies. This is especially relevant for the clinical management because fibrosis in the main prognostic indicator in patients with NAFLD [6,7]

In line with previous results[8], we observed that progression of fibrosis did not require the presence of baseline NASH, despite NASH was related to more severe fibrosis at baseline and associated with progression of fibrosis. However, follow-up liver biopsy was not systematically performed in patients without baseline NASH. Therefore, an indication bias may have led to selection of patients with worsening metabolic status, liver enzymes, and noninvasive predictors of liver damage. Indeed, in about half of the cases, progressors developed NASH at follow-up. Notwithstanding, data suggest that preventive strategies and clinical trials should not only focus on individuals with histological severe inflammation and hepatocellular ballooning. It is therefore imperative to identify other risk factors and disease mechanism contributing to disease evolution.

One may be represented by T2D and the severity of insulin resistance, which, in line with data reported in a previous UK study evaluating a comparable number of patients[8], was identified as a predictor of fibrosis progression in the present cohort. There is ample literature on the role of insulin resistance and hyperglycemia in the progression of NAFLD[3,31,32]. However, the mechanisms linking metabolic abnormalities with fibrosis progression independently of NASH and inflammation, even in patients with normal liver enzymes[33], need further clarification. Indeed, this may represent a different form of NAFLD progressing to hepatic complications by inflammation-independent pathways, for which specific noninvasive biomarkers and therapeutic approaches should be developed.

Remarkably, fibrosis progression was associated with the length of the observation, being more frequent in patients observed for an average 5-years period. This supports the EASL-EASD-EASO guidelines that suggest to consider re-biopsy after this timeframe[29]. We also identified the absolute value of APRI score, not reported in a previous cohort[8], as the most reliable predictor of fibrosis progression. These results need however replication.

An important novel aspect that could be addressed in the present database, but could not be evaluated in a previous meta-analysis and in a recent study [8,9], was the impact of therapies on FPR. A major finding was that patients in treatment with RAS inhibitors had slower FPR, and lower likelihood of fibrosis progression. Furthermore, use of RAS inhibitors resulted independently associated with lack of fibrosis progression also at logistic regression analysis considering variables associated at univariate analysis, providing an independent confirmation of this association by an alternative approach. This is in line with accumulating evidence indicating that angiotensin is involved in hepatic stellate cells activation, while RAS inhibitors contrast fibrogenesis in experimental models[13–20], and their use was associated with protection from fibrosis in cross-sectional studies in patients with NAFLD and kidney disease[23,24]. Interestingly, the protective effect of RAS inhibitors was particularly evident in patients with T2D, but not in patients with arterial hypertension in general. This would suggest that activation of RAS represents a specific feature driving both kidney [34] and hepatic fibrogenesis in T2D. Noteworthy, activation of the neuro-vegetative system is associated with induction of RAS activation, and the two have a synergic role in the pathogenesis of arterial hypertension and kidney dysfunction in overweight individuals[35]. More widespread use of RAS inhibitors in patients with baseline NASH may have contributed to the slow FPR observed in this subgroup despite severe histological activity. On the other hand, we could not detect any significant effect of statins [2,36], or iron depletion [37] on FPR.

Limitations of this study include that the sample size did not allow to test the effect of specific therapeutic molecules on FPR, and to test for a dose-response effect. Furthermore, findings may not be extended to other ethnic groups and populations with different genetic and lifestyle risk factors. This study does not report results of a randomized trial, therefore the association of therapeutic approaches with liver disease evolution should be considered as hypothesis generating, and not as a proof of efficacy. In addition, treatment of T2D has recently evolved, but in the present cohort only a few patients were taking oral hypoglycemic drugs other than metformin. Larger collaborative studies are needed to better characterize the impact of new as well as “old” pharmacological treatments on the progression of liver disease in NAFLD.

In conclusion, our data confirm that NASH is not required for fibrosis progression in NAFLD, whereas T2D seems to drive fibrogenesis independently of hepatic inflammation. Most importantly, we could show for the first time in a prospective evaluation of retrospectively collected data by two independent approaches that use of RAS inhibitors may contrast fibrosis progression especially in high-risk patients affected by T2D. These findings further reinforce the indication of use RAS inhibitors to treat arterial hypertension or initial kidney disease in T2D patients in the presence of NAFLD.

## Supporting Information

S1 File Supplementary methods. (DOCX)

S2 File Supplementary references. (DOCX)

S1 Table Impact of baseline histological features of FPR in 118 Italian patients with NAFLD. (DOCX)

S2 Table Association of the changes of clinical variables during follow-up with fibrosis progression. (DOCX)

S3 Table Independent predictors of fibrosis progression at multivariate logistic regression analysis in 108 Italian patients with NAFLD without F4 fibrosis at baseline. (DOCX)
