# Supplementary material for: Renin-Angiotensin System Inhibitors, Type 2 Diabetes and Fibrosis Progression: An Observational Study in Patients with Nonalcoholic Fatty Liver Disease
Source: PLoS One. 2016 Sep 20;11(9):e0163069. doi: 10.1371/journal.pone.0163069 (PMC5029872; doi:10.1371/journal.pone.0163069)
Supplement: S1 File — (DOCX) [file pone.0163069.s001.docx]

Supplementary methods

*Clinical and Laboratory Assessment*

Clinical and anthropometric data were collected at the time of liver biopsy. Body mass index (BMI) was calculated on the basis of weight in kilograms and height in meters. The diagnosis of type 2 diabetes was based on the revised criteria of the American Diabetes Association, using a value of fasting blood glucose ≥126 mg/dl on at least two occasions ^1^. In patients with a previous diagnosis of T2D, current therapy with insulin or oral hypoglycemic agents was documented.

A 12-hour overnight fasting blood sample was drawn at the time of biopsy to determine serum levels of ALT, total cholesterol, HDL-cholesterol, triglycerides, plasma glucose and insulin concentrations. IR was assessed by homeostasis model assessment (HOMA) , using the following equation: Insulin resistance (HOMA-IR) = Fasting insulin (μU/mL) x Fasting glucose (mmol/L)/22.5 ^2^. *PNPLA3* I148M variant was determined by Taqman assays as previously described in 89 patients for whom DNA samples and consent were available ^3, 4^. Non-invasive scores of fibrosis in NAFLD were calculated as previously described ^5^. Pharmacological history was recorded both at baseline and follow-up liver biopsy. Use of RAS inhibitors was defined in the presence of baseline history of at least 6-months use of ACE-I/ARBs.

*Variables considered in stepwise regression analysis (Table 5)*

Variables considered in stepwise regression analysis to identify predictors of fibrosis progression rate (FPR):

- Clinical features at baseline and follow-up: age, sex, BMI, glucose, total cholesterol, HDL cholesterol, triglycerides, ALT, AST, GGT, ferritin, platelets, APRI score, FIB-4 score, NFS score.
- Histological features at baseline and follow-up: steatosis grade, ballooning grade, necro-infammatory grade.
- At baseline and/or follow-up, presence of: type 2 diabetes, arterial hypertension, NASH.
- At baseline and/or follow-up, treatment with: renin-angiotensin axis (RAS) inhibitors (ace-inhibitors or angiotensin receptor antagonists), beta-blockers, diuretics, calcium antagonists, statins, omega-3 fatty acids, vitamin E, iron depletion therapy.

Among selected variables, if collinearity was detected we included in the final model only the variable most strongly associated with FPR.
