# Supplementary material for: Renin-Angiotensin System Inhibitors, Type 2 Diabetes and Fibrosis Progression: An Observational Study in Patients with Nonalcoholic Fatty Liver Disease
Source: PLoS One. 2016 Sep 20;11(9):e0163069. doi: 10.1371/journal.pone.0163069 (PMC5029872; doi:10.1371/journal.pone.0163069)
Supplement: S2 File — (DOCX) [file pone.0163069.s002.docx]

SUPPLEMENTARY REFERENCES

1. Third Report of the National Cholesterol Education Program (NCEP) Expert Panel on Detection, Evaluation, and Treatment of High Blood Cholesterol in Adults (Adult Treatment Panel III) final report. Circulation. 2002;106(25):3143-421.

2. Bonora E, Targher G, Alberiche M, et al. Homeostasis model assessment closely mirrors the glucose clamp technique in the assessment of insulin sensitivity: studies in subjects with various degrees of glucose tolerance and insulin sensitivity. Diabetes Care. 2000;23(1):57-63.

3. Mancina RM, Dongiovanni P, Petta S, et al. The MBOAT7-TMC4 Variant rs641738 Increases Risk of Nonalcoholic Fatty Liver Disease in Individuals of European Descent. Gastroenterology. 2016.

4. Dongiovanni P, Donati B, Fares R, et al. PNPLA3 I148M polymorphism and progressive liver disease. World J Gastroenterol. 2013;19(41):6969-78.

5. Angulo P, Hui JM, Marchesini G, et al. The NAFLD fibrosis score: a noninvasive system that identifies liver fibrosis in patients with NAFLD. Hepatology. 2007;45(4):846-54.
