# Supplementary material for: Renin-Angiotensin System Inhibitors, Type 2 Diabetes and Fibrosis Progression: An Observational Study in Patients with Nonalcoholic Fatty Liver Disease
Source: PLoS One. 2016 Sep 20;11(9):e0163069. doi: 10.1371/journal.pone.0163069 (PMC5029872; doi:10.1371/journal.pone.0163069)
Supplement: S1 Table — (DOCX) [file pone.0163069.s003.docx]

Table S1. Impact of baseline histological features of FPR in 118 Italian patients with NAFLD.

| Histological feature | Estimate | p value | Adjusted p value* |
| --- | --- | --- | --- |
| Steatosis | -0.006±0.004 | 0.23 | 0.13 |
| Necro-inflammation | -0.001±0.006 | 0.84 | 0.40 |
| Ballooning | -0.005±0.005 | 0.78 | 0.28 |

* Adjusted for age, sex, BMI, and type 2 diabetes.
