# Supplementary material for: Renin-Angiotensin System Inhibitors, Type 2 Diabetes and Fibrosis Progression: An Observational Study in Patients with Nonalcoholic Fatty Liver Disease
Source: PLoS One. 2016 Sep 20;11(9):e0163069. doi: 10.1371/journal.pone.0163069 (PMC5029872; doi:10.1371/journal.pone.0163069)
Supplement: S2 Table — (DOCX) [file pone.0163069.s004.docx]

Table S2. Association of the changes of clinical variables during follow-up with fibrosis progression.

| Clinical features | p value* |
| --- | --- |
| BMI, Kg/m^2^ | 0.13 |
| T2D, new | 0.67 |
| Glucose, mg/dl | 0.94 |
| Total cholesterol, mg/dl | 0.31 |
| HDL cholesterol, mg/dl | 0.93 |
| Triglycerides, mg/dl | 0.20 |
| Arterial hypertension, new | 0.094 |
| ALT, IU/ml | 0.28 |
| AST, IU/ml | 0.28 |
| GGT, IU/ml | 0.75 |
| Ferritin (ng/mL) | 0.35 |
| Platelets (x10^9/L) | 0.92 |
| APRI score | 0.12 |
| FIB4 score | 0.48 |
| NFS | 0.58 |

* Adjusted for length of follow-up.
