# Supplementary material for: Renin-Angiotensin System Inhibitors, Type 2 Diabetes and Fibrosis Progression: An Observational Study in Patients with Nonalcoholic Fatty Liver Disease
Source: PLoS One. 2016 Sep 20;11(9):e0163069. doi: 10.1371/journal.pone.0163069 (PMC5029872; doi:10.1371/journal.pone.0163069)
Supplement: S3 Table — (DOCX) [file pone.0163069.s005.docx]

Table S3. Independent predictors of fibrosis progression at multivariate logistic regression analysis in 108 Italian patients with NAFLD without F4 fibrosis at baseline.

| Histological feature | OR | 95% c.i. | p value |
| --- | --- | --- | --- |
| Follow-up, months | 1.02 | 1.00-1.03 | 0.004 |
| RAS inhibitors, yes | 0.09 | 0.01-0.51 | 0.004 |
| APRI at follow-up, units | 8.98 | 1.66-70.0 | 0.010 |
| NASH at baseline, yes | 3.99 | 1.37-12.8 | 0.011 |
| T2D at baseline, yes | 3.93 | 1.05-15.6 | 0.042 |

RAS: renin angiotensin system axis; T2D: type 2 diabetes.
